# Ultra-low Doses of Follicle Stimulating Hormone and Progesterone Attenuate the Severity of Polycystic Ovary Syndrome Features in a Hyperandrogenized Mouse Model

**Published:** 2017

**Authors:** Irene Tessaro, Silvia Modina, Valentina Lodde, Giulia Sivelli, Federica Franciosi, Laura Terzaghi, Patrizia Luchini, Cristiano Rumio, Alberto Maria Luciano

**Affiliations:** 1- Reproductive and Developmental Biology Laboratory, Department of Health, Animal Science and Food Safety, University of Milan, Milan, Italy; 2- Istituto di Ricovero e Cura a Carattere Scientifico (IRCCS), Istituto Ortopedico Galeazzi, Milan, Italy; 3- Interdepartmental Centre for the Study of Biological Effects of Nano-Concentrations (CREBION), University of Milan, Milan, Italy; 4- Department of Pharmacological and Biomolecular Sciences, University of Milan, Milan, Italy

**Keywords:** Follicle cyst, FSH, Mouse, Polycystic ovary syndrome, Progesterone, Sequential kinetic activation, Ultra-low dose

## Abstract

**Background:**

Polycystic-ovary syndrome (PCOS) is a reproductive illness characterized by hyperandrogenism and anovulation. Using hyperandrogenized mice, it was demonstrated that the oral administration of incremental dose of follicle stimulating hormone (FSH) attenuated some of PCOS characteristics. This work aimed to study the effect of ultra-low doses of combined FSH and progesterone orally administered on PCOS murine model. Moreover, the effect of sequential kinetic activation of administered hormones was tested.

**Methods:**

Thirty-two female mice were used as animal model (four groups of eight animals each). Mice were hyperandrogenized by injection of dehyidroepiandrosterone diluted in sesame oil. Control group received only oil. Simultaneously, each animal daily received per os an activated or a not-activated combination of FSH (0.44 *pg*) plus progesterone (0.44 *pg*) or saline solution as control. Serum testosterone, estradiol, progesterone and luteinizing hormone were analyzed as endocrine markers and a morphological study of antral follicle was conducted. Data were analyzed by one-way ANOVA, followed by multiple comparison test. The p<0.05 was considered significant.

**Results:**

Dehyidroepiandrosterone treatment increased both estradiol and progesterone serum levels, besides testosterone, while reduced luteinizing hormone (p<0.05); histological examination revealed an increase of cystic follicles (p<0.05). Irrespective of activation, the combined FSH and progesterone treatments restored estradiol level (p>0.05 *vs.* control group) and reduced cystic signs in the follicles (p<0.05 *vs*. dehyidroepiandrosterone treatment).

**Conclusion:**

This study indicate that ultra-low doses of FSH and progesterone orally administrated can reduce the sternness of PCOS in the mouse model and open a route for the study of innovative approaches for PCOS treatment.

## Introduction

Polycystic Ovary Syndrome (PCOS) is a wide-spread reproductive disorder described by hyperandrogenism, polycystic ovaries, and anovulation (1). When the normal ovarian function is disturbed, it results in multiple small cysts (2, 3). A nearly widespread finding in PCOS is an augmented gonadotropin-releasing hormone (GnRH) pulse frequency, determining an excess of luteinizing hormone (LH) over follicle stimulating hormone (FSH) (4, 5). This endocrinologic derangement may be associated with impaired hypothalamic sensitivity to P4 (6). The increased LH successively promotes theca cell androgens production, while the relative FSH deficiency interferes with granulosa cell aromatization to estrogens and impairs follicle maturation and ovulation (7).

Moreover, 60–80% of PCOS patients display insulin resistance, which can contribute to an excess of insulin in the ovaries, that in turn can enhance the responsiveness of theca cells to LH, producing a surplus of androgens at that site (8, 9). A “vicious cycle” of excess androgen production is thus generated.

The management of PCOS patients is usually focused on symptoms, typically menstruation related disorders, androgen-related symptoms, or infertility (10). Ovarian stimulation with gonadotropins is one of the therapies for PCOS infertile patients; however, because of the large number of small antral follicles that are sensitive to FSH (11), women with PCOS have a higher risk of multiple pregnancy and ovarian hyperstimulation syndrome (OHSS; (12)) in response to FSH treatment (13). To minimize this possibility, protocols for administration of FSH at low-dose have been suggested (14). Nevertheless, the consensus on infertility treatment related to PCOS (15, 16) concluded that although low-dose FSH protocols are effective in achieving ovulation, further refinement is needed to better control the safety of the low-dose FSH protocols. In this contest, ultra-low dose of FSH regiment was proposed, achieving significant clinical results (17) and demonstrating the current interest in developing new protocol with an excellent safety profile.

Animal models that mimic the traits of PCOS could facilitate research, leading both to an understanding of the pathogenesis and identifying new and more effective treatments. Androgen-treated rodents have been broadly used as models to study both reproductive and metabolic deficiencies of PCOS (18–20). Using dihydroepiandrosterone (DHEA) hyperandrogenized mice as a model, it was recently demonstrated that the oral administration of weekly incremental dose of FSH in a low-dose step-up protocol could mitigate some of the characteristics of PCOS in the murine model (21), suggesting a novel and more convenient therapeutic approach. Considering those results, the aim of this work was to investigate endocrine and morphological effects of a combination of ultra-low doses of FSH and P4 orally administered to a hyperandrogenized mice model. Since activation is one of the processes to enhance pharmaceutical preparations efficacy (22, 23), the effect of sequential kinetic activation (SKA) of the administered hormones was also tested.

## Methods

Except for those specifically mentioned, all chemicals were purchased from Sigma Chemical Company (St. Louis, MO, USA).

### Animals:

Female Balb/c mice (Mus musculus; strain code: 028; specific pathogen free; (Charles River Laboratories Italia s.r.l., Calco (LC), Italy)), were housed in groups of four and given twelve days to acclimate to the housing facility. Environmental conditions were a temperature of 21± 2°*C*, humidity of 50%±10%, and a 12:12 light: dark cycle. The cages and the housing room were cleaned before the animals were accommodated, then cleaning and disinfection were performed twice a week. Animals were housed in 160×270×370 *mm* (L×W×H) cages and fed ad libitum with a standard pelletized diet (standard mouse diet 4RF18, Mucedola srl, Milan, Italy) and purified water. During housing, animals were monitored twice daily for health status. No adverse events were observed. All procedures were carried out in a standard and certified animal care facilities of University of Milan. The experimental protocol was approved by the University of Milan Ethics Committee in accordance to the European Directive 2010/63/EU revising Directive 86/609/EEC on the protection of animals used for scientific purposes. All sections of this report adhere to the ARRIVE Guidelines for reporting animal research (24).

### Hyper-androgenization and hormonal treatment:

All experiments were performed on 40 day-old mice as previously described (21). Hyper-androgenized mice received daily a subcutaneous (SC) injection of DHEA (1.2 *mg/mouse/day*, derived from 6 *mg*/100 *g* body weight (25)) dissolved in 0.1 *ml* sesame oil for 20 consecutive days. To study the effect of oral administration of FSH and P4 on PCOS-induced animals, four groups of eight mice each were used (Table 1), similarly to the previous study (21). The thirty-two mice were randomly allocated to four groups (eight animals per group). Based on previous knowledge on activated blends (22, 23), the ultra-low dose hormones solutions were prepared in two different formulations: an activated solution (FSHP4), triggered by a standardized method of pharmaceutical preparation known as SKA (GUNA S.p.a, Milan, Italy), and a non-activated solution (NA-FSHP4). The activated solutions underwent a shaking process, characterized by the following parameters: vertical shaking; 10 centimeters motion range; shaking speed corresponding to 100 oscillations in 10 seconds (26, 27).

**Table 1.** Experimental design

**Treatments**	**Sub-cutaneous injection (Total volume=0.1 *ml*)**	**Per os administration (Total volume=0.1 *ml*)**
**CTRL**	Sesame oil	Saline solution
**DHEA**	DHEA (1.2 *mg*)	Saline solution
**DHEA+FSHP4**	DHEA (1.2 *mg*)	activated FSH (0.44 pg)+activated P4 (0.44 *pg*)
**DHEA+NA-FSHP4**	DHEA (1.2 *mg*)	not activated FSH (0.44 pg)+not activated P4 (0.44 *pg*)

Daily sub-cutaneous injection and per os administration for each experimental group composed of 8 mice. The duration of the treatment was 20 days

Therefore, simultaneous to DHEA treatment, each mouse of the first group (FSHP4) received daily 0.44 *pg* of activated FSH plus 0.44 *pg* of activated P4, which correspond to 1.17×10^7^ molecules of FSH and 8.42×10^8^ molecules of P4. in a total volume of 0.1 *ml* of saline solution, administered directly into the stomach via oral gavage once a day for 20 consecutive days. Each mouse of a second group (NA-FSHP4) received daily 0.44 *pg* of not activated FSH plus 0.44 *pg* of not activated P4 in a total volume of 0.1 *ml* of saline solution by oral gavage, as previously described. A third group (DHEA) was treated with DHEA subcutaneously and with saline solution by oral gavage. The last group received daily sesame oil subcutaneously and 0.1 *ml* of saline solution by oral gavage and served as control (CTRL). The subcutaneous injections, right after the oral administrations, were performed every day at 2:00 *pm*, in the same room of housing. Mice were treated following the same order. Animal body weight was measured at the beginning and at the end of the 20-day treatments.

### Hormonal analysis of serum:

At the end of the treatments, blood samples were collected from each mouse and the serum was separated and analyzed, as previously described (21). Briefly, testosterone (T) quantification was performed through a competitive inhibition enzyme immunoassay technique (Mouse Testosterone ELISA Kit, CSB-E05101 *m*, China), whereas P4, estradiol (E2) and LH levels were measured using a magnetic bead immunoassay based on Luminex Multiplex System (Merck Millipore, Germany), according to the manufacturer’s instructions; respectively, P4 and E2 were analyzed by the “Steroid/Thyroid Hormone Magnetic Bead Panel” (#STTHMAG-21K) and the “Mouse Pituitary Magnetic Bead Panel” (#MPTMAG-49K) was used for LH.

### Morphological evaluation of ovaries:

The ovaries were collected immediately after the sacrifice and fixed in 10% neutral buffered formalin (Bio-Optica, Milano, Italy) over night. The ovaries were processed and serially sectioned as previously described (21). Briefly, slices were stained with haematoxylin and eosin (DDK Italia, Vigevano, Italy) and finally analyzed under light microscopy to assess follicles diameter and morphological features (28). To select a representative follicular population, one slice every 150 *μm* was considered, and were used to identify the antral follicles to be analyzed. Follicle diameter was calculated as the mean distance between opposite basal membrane portions, whereas the wall thickness was calculated as the sum of theca interna and granulosa cell layers. According to follicle diameter, the follicular population was divided in two classes: early-small antral follicles (150–300 *μm*) and large antral follicles (>300 *μm*) (29–31). The presence of morphological cystic features (32–36) was recorded for each antral follicle. Two investigators independently and blindly performed morphological analysis.

### Immunoexpression of aromatase cytochrome P450:

The expression of cytochrome P450 aromatase (P450 arom) was evaluated by immunohistochemistry procedures, as previously described (21). Briefly the protein was detected by incubation with 4 *μg/ml* of polyclonal goat anti-CYP19 (CYP19 (C-16): sc-14245, Santa Cruz Biotechnologies, Inc, USA) and then with Alexa Fluor 488-labeled rabbit anti-goat IgG. Negative controls were performed by omitting the primary antibody. Nuclei were counterstained by 1 *μg/ml* 4′,6-diamidino-2-phenylindole (DAPI) and follicles were analyzed by epifluorescence microscopy (Eclipse E600, Nikon Corp., Tokyo, Japan) at a magnification of 200X–400X.

### Statistical analysis:

Statistical analyses were performed using Prism GraphPad (GraphPad Software, version 6.0f, San Diego, CA, USA). Each animal was considered as an experimental unit. All the animals (thirty-two mice) were included in all the analysis. Data were analyzed by one-way ANOVA, followed by Fisher’s Least Significance Difference (LSD) multiple comparison test. Regardless of test, p-values<0.05 were considered significant.

## Results

### Body weight changes:

The effect of hormonal treatments on the body mass was evaluated by weighting mice. Before treatments, the weight of animals was not statistically different between groups (Table 2). Mice gained weight during the experiment, but DHEA treated animals gained about 10% more than the controls (Table 2).

**Table 2.** Effect of different hormonal treatments on body weight of mice

**Treatments**	**Weight (*gr*) on day 0**	**Weight (*gr*) on day 21**
**CTRL**	15.5±0.42^a^	19.38±0.37^b^
**DHEA**	16.63±0.46^a^	21.38±0.37^c^
**DHEA+FSHP4**	16.75±0.41^a^	22.13±0.48^c^
**DHEA+NA-FSHP4**	15.75±0.67^a^	21.13±0.81^c^

Data were expressed as mean±SEM and were analyzed by one-way ANOVA, followed by Fisher’s LSD multiple comparison test; different letters indicate significant differences between groups (p<0.05)

### Hormonal analysis:

The effect of ultra-low doses administration of FSH and P4 on the serum hormonal profiles was evaluated using immunoassay techniques and results are summarized in table 3. Testosterone and P4 concentrations were statistically higher in all the PCOS-induced animals (DHEA treated) compared to CTRL. No differences were observed in animals treated with FSH and P4. DHEA treatment significantly increased serum E2 concentration compared to CTRL, and both FSHP4 and NA-FSHP4 treatments restored an E2 level statistically similar to the CTRL. DHEA alone or with FSHP4 caused a significant decrease of LH concentration compared to CTRL.

**Table 3.** Effect of different hormonal treatments on testosterone (T), progesterone (P4), estradiol (E2) and luteinizing hormone (LH) serum concentration

**Treatments**	**T (*ng/ml*)**	**P4 (*ng/ml*)**	**E2 (*ng/ml*)**	**LH (*ng/ml*)**
**CTRL**	0.21±0.01^a^	12.75±3.09^a^	1.43±0.15^a^	0.53±0.06^a^
**DHEA**	0.89±0.14^b^	37.1±3.72^b^	2.53±0.24^b^	0.19±0.02^b^
**DHEA+FSHP4**	0.92±0.2^b^	30.45±3.44^b^	1.65±0.21^a^	0.15±0.01^b^
**DHEA+NA-FSHP4**	0.84±0.13^b^	36.51±3.62^b^	1.36±0.1^a^	0.16±0.01^b^

Data were expressed as mean±SEM and were analyzed by one-way ANOVA, followed by Fisher’s LSD multiple comparison test. In each column, different letters indicate significant differences between treatments (p<0.05)

### Morphological evaluation of ovaries:

The potential role of the oral administration of ultra-low doses of FSH and P4 on follicle development in DHEA-treated animals was evaluated by monitoring the number of follicles 150–300 *μm* in diameter and of follicles >300 *μm* in diameter. DHEA treatment significantly reduced the number of small follicles per ovary, even if both FSHP4 and NA-FSHP4 attenuated DHEA’s action (Table 4). On the other hand, DHEA increased the number of large follicles per ovary and both the formulations of FSHP4 were able to weaken DHEA’s effect, decreasing the large follicle population although not significantly (Table 4).

**Table 4.** Effect of different hormonal treatments on the wall thickness, on the number of antral follicles per ovary and the percentage of cystic/atretic follicles

**Treatments**	**Wall thickness (*μm*)**		**Number of follicles/ovary**		**Percentage of cystic follicles**	

	**Small follicles (150–300 *μm*)**	**Large follicles (>300 *μm*)**	**Small follicles (150–300 *μm*)**	**Large follicles (>300 *μm*)**	**Small follicles (150–300 *μm*)**	**Large follicles (>300 *μm*)**
**CTRL**	28.83±1.32	64.87±4.71^a^	12.63±1.18^a^	0.75±0.36^a^	26.82±5.66	0.25±0.16^a^
**DHEA**	32.21±2.14	47.06±2.18^b^	6.37±1.4^b^	7.62±1.13^b^	39.05±13.14	67.23±3.04^b^
**DHEA+FSHP4**	33.32±2.7	47.39±1.75^b^	10.25±0.99^ab^	5.5±0.84 ^b^	24.11±6.79	42.76±6.01^c^
**DHEA+NA-FSHP4**	28.77±1.41	50.44±2.25^b^	9.37±1.8^ab^	5.1±0.85^b^	16.67±5.32	34.06±9.41^c^

Data were expressed as mean±SEM and were analyzed by one-way ANOVA, followed by Fisher’s LSD multiple comparison test. In each column, different letters indicate significant differences between groups (p<0.05)

In the large follicles (>300 *μm*), DHEA treatment induced a decrease of theca and granulosa cells layers thickness, irrespective of other hormonal administration (Table 4). No differences were observed in small follicles (150–300 *μm*).

**Figure 1.**
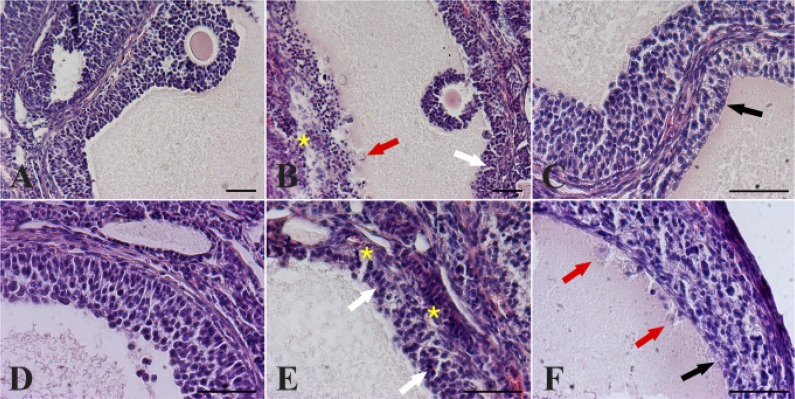
Effect of different hormonal treatment on the percentage of follicles presenting atretic/cystic signs. Descriptive images of peculiar morphological changes in antral follicle walls of ovaries isolated from controls (A, D) compared to DHEA-treated mice (B, C, E, F), stained with haematoxylin and eosin. In the control (A, D), theca externa, theca interna, basal membrane and granulosa cells layers appear normal. Cystic features are described by presence of spherical piknotic nuclei (B), loss of mural granulosa cells architecture (B and E, white arrows), depletion of basal membrane (B and E, asterisks), macrophages in the cystic fluid (B and F, red arrows), thin and elongated epithelioid cells in the inner surface of the wall (C and F, black arrows) and reduction of granulosa cells layers (C). Bars=50 *μm*

Features of follicle atresia and cystic formations were described (Figure 1): cell pyknosis, interruption of the basement membrane and thecal layers’ disruption, reduction of granulosa cells layers, presence of elongated epithelioid cells in the surface lining the follicle wall. Finally, the invasion of blood cells and macrophages in the wall of the follicle as well as in the cystic fluid were observed (Figure 1). DHEA significantly increased the incidence of morphological cystic features in antral follicles with a diameter >300 *μm* per ovary (Table 4); however, the oral administration of either activated or not activated solution of FSH and P4 decreased the percentage of atretic/cystic features in large follicles in DHEA-treated mice, even if it was still greater than CTRL. Finally, in the 150–300 *μm* follicle population, FSHP4 and NA-FSHP4 oral administration reduced the percentage of atretic/cystic follicles on total follicular population compared to DHEA treatment alone (Table 4).

### Localization of aromatase cytochrome P450:

**Figure 2.**
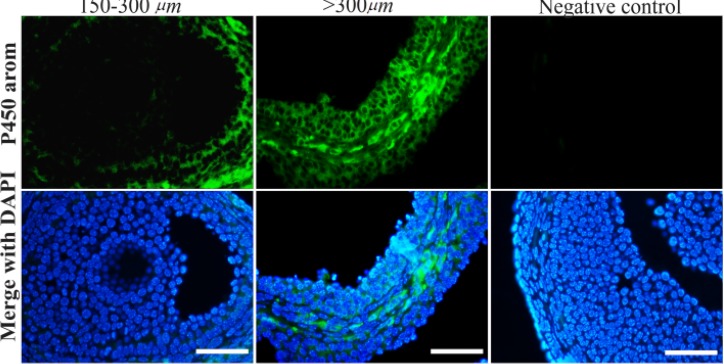
Immunohistochemical localization of aromatase cytochrome P450. No or weak aromatase protein was noticed in early-small antral follicles (150–300 *μm*; on the left), as a positive staining was evident in large antral follicles (>300 *μm*; in the middle). Control specimens did not exhibit any positive staining (on the right). Scale bars=50 *μm*

The signal for P450 arom was always absent or weak in 150–300 *μm* early-small antral follicles, regardless of the the treatment (Figure 2) while it was present in the cytoplasm of granulosa cells in >300 *μm* large antral follicles. In particular, P450 arom was concentrated inside the cytoplasm of mural cells lying on the basal membrane (Figure 2) with negligible or no staining in cumulus cells. No signal was observed in control sections.

## Discussion

PCOS is the most common endocrinopathy of women of reproductive age (37). Its high incidence has attracted significant public consideration (>1.5×10^6^ sites dedicated to the syndrome (38)). Moreover, identification and management of PCOS have been estimated to cost $4 billion annually only to the USA healthcare system (39). Similar data are not available for Europe. Despite its occurrence, there is no agreement on long-term management of women with PCOS and conventional pharmacological treatments are symptom focused and often have side effects. Therefore, the need for efficient treatment for PCOS is inducing the diffusion of research studies on innovative approaches concurrent to conventional medical management of the syndrome (40). In this context, the present results confirm and expand our previous findings on the oral administration of low doses of FSH (21). The present study demonstrates that also ultra-low doses can ameliorate several aspects related to hyperandrogenism in a PCOS-induced mice model. In fact, the administration of the hormone combination restored the physiological serum level of estradiol and diminished the percentage of cystic follicles. However, the positive effects of the hormonal treatment were observed regardless of the SKA protocol.

The interest in activated blends is growing in different fields, mostly pharmaceutical technology, though the mechanism underlying the efficacy is still unknown. To test the contribution of activation process, the effect of the activated and not-activated preparation was analyzed in the same experimental condition. Although other authors found that activation protocol highly increased efficacy of activated solutions (26, 27, 41–43), our data showed that no differences are present regarding the considered parameters between the SKA-activated and the not-activated preparation.

In agreement with literature and with our previous work, present results confirmed DHEA-treated mice as an appropriate model to study PCOS syndrome, replicating most common clinical features of this reproductive disorder (44). Most of the animal models are based on induction of hyperandrogenism, especially by DHEA administration, an androgen often increased in women with PCOS (45). The dose commonly used in mice (6 *mg*/100 *g* body weight) provokes infertility and the formation of atretic follicles and follicular cysts in the ovaries (46–48). As previously pointed out (21), DHEA treatment increased animal weight and the serum concentration of steroid hormones, in addition to the cystogenesis induction. Despite LH over-production is one of the peculiar traits of PCOS in women (6), in these experiments, the decrease of LH in the serum of DHEA treated mice was verified, as described beforehand (21).

The administration of gonadotropin has been broadly used for ovulation induction in patients with PCOS, but the development of multiple follicles and consequently multiple pregnancies and/or OHSS emerged as the side effects (49). Due to the social and medical implication of a multiple gestation, the implementation of low-dose treatment programs arose. Nevertheless, the recent agreement on infertility treatment related to PCOS (15) concluded that although low-dose FSH protocols are effective in achieving ovulation in women with PCOS, further improvements are essential to guarantee the safety of this innovative treatments.

In the present work, the treatment of PCOS-induced mice with very high dilution of FSH and P4 was proposed. Therefore, 0.44 *pg/die* of FSH was administered, which is considerably lower than the dose commonly administered in low dose clinical therapy for anovulatory infertility (50 *IU* in women recalculated according to the average weight to 0.02 *IU* in the mouse, corresponding to 2 *ng*).

The ultra-low dose of FSH was daily administered simultaneously to P4. This hormone has been often used as the luteal-phase support in ovulation induction. As previously suggested, the endocrinologic disorders that result in abnormal follicular development and frequent anovulation in PCOS patients may be associated with impaired hypothalamic sensitivity to P4 (6). Granulosa cells of women with PCOS appear to be incompetent to physiological P4 secretion after luteinization if ovulation is achieved (50). Therefore, women with PCOS may essentially benefit from P4 supplementation in the luteal phase (51). Progesterone administration decreases LH pulse frequency (52), a finding observed naturally in the luteal phase during ovulatory cycles in these women (53). Progesterone may also provide a variety of beneficial effects for women with PCOS, who often exhibit delayed endometrial development (54, 55). In addition to the general action of limiting the epithelial proliferative effects of estrogen and inducing decidualization, P4 down-regulates endometrial androgen receptors, the expression of which is elevated in women with PCOS (56). In the clinical practice, P4 is normally administered vaginally at a concentration of 200 *mg* twice a day (51). Our data indicated that the oral administration of an ultra-low dose of P4 (0.44 *pg/die*) could cooperate in restoring several peculiar features of PCOS in our model. However, in the present preliminary work, the simultaneous administration of P4 and FSH attenuated some of the PCOS traits in the hyperandrogenized murine model to a similar extent of previous study where low dose of FSH was administered alone (21). For this reason, further studies are needed to better define the specific role of P4 in the softening of the PCOS phenotype severity in the mouse model.

First, the administration of FSHP4 restored the E2 serum level. This phenomenon appears to reflect the functionality of granulosa cells, since granulosa cells from anovulatory women with PCOS hyper-secrete E2, compared to size-matched follicles from normal ovaries (57). The increase of serum E2 revealed the intensification of follicular atresia processes in DHEA-treated animals, while oral treatment with ultra-low doses of FSH and P4 was able to reduce E2 concentration (Table 3). This decline probably reflects the partial normalization of follicular development; both FSHP4 and NA-FSHP4 treated ovaries presented a decrease of atretic and cystic status in both growing and large antral follicles and in the meanwhile the development of a new population of small growing healthy follicles, which synthetize and secrete less E2 (58, 59) (Table 4). This hypothesis is confirmed by the immunohistochemical study which underlines the lack of P450 arom in the small follicles cells different from the larger follicles. Thus, the observed significant reduction in serum E2 in the FSHP4 treated animals is associated with the reduction of the number of large antral follicles typically expressing aromatase.

Furthermore, in our study, ultra-low doses of FSH and P4 orally administered reduced the presence of atresia and attenuated the cystic formation, maintaining the viability of large antral follicles. To the best of our knowledge, the mechanism is not completely understood, even if another study in a Guinea pig reported that FSH treatment diminishes the ovarian cyst formation (60).

Thus, FSHP4 administration was able to partially reinstate the physiological status of ovarian follicles taking in consideration both morphological and functional characteristics. During estrous cycle, FSH stimulates proliferation of granulosa cells in primary follicles, and once a follicle reaches a diameter of approximately 150 *μm*, FSH induces antrum formation and aromatase activity in the granulosa cells with a gradual increase in estradiol synthesis (61, 62). Additionally, our results further support the feasibility of administering effective gonadotropin treatment orally.

Gonadotropins are conventionally delivered via routes such as intramuscular, subcutaneous, or intravenous injections due to their reduced oral bioavailability since peptides can be readily degraded and pass poorly through the intestinal mucosa (63, 64). Interestingly, our results indicate that orally administrated FSH, in combination to P4, can reduce the severity of PCOS in the hyperandrogenized mouse model system. On the other hand, it is unlikely that intact FSH is transferred from the gastrointestinal tract into the circulation. The identification of the mechanism by which the orally administered FSH acts has yet to be clarified, and this is a limit to the present study. However, since an oral route would be most desirable to obtain maximum patients compliance, further investigations are strongly encouraged.

## Conclusion

Overall, in the present study, the effect of supplementation of ultra-low doses of FSH in addition to P4, orally supplemented, was characterized in a hyperandrogenized murine model of PCOS. Our results demonstrated that the treatment can diminish the harshness of some PCOS features, indicating a novel and intriguing way that should be examined in depth.
